# Database combinations to retrieve systematic reviews in overviews of reviews: a methodological study

**DOI:** 10.1186/s12874-020-00983-3

**Published:** 2020-06-01

**Authors:** Käthe Goossen, Simone Hess, Carole Lunny, Dawid Pieper

**Affiliations:** 1 Institute for Research in Operative Medicine (IFOM), Faculty of Health, School of Medicine, Witten/Herdecke University, Ostmerheimer Str. 200, 51109 Cologne, Germany; 2 Department of Anesthesiology, Pharmacology and Therapeutics, Faculty of Medicine, Cochrane Hypertension Review Group and the Therapeutics Initiative, University of British Columbia, 2329 West Mall, Vancouver, BC V6T 1Z4 Canada

**Keywords:** Review methods, Overview of reviews, Umbrella review, Search strategy, Databases, Systematic reviews

## Abstract

**Background:**

When conducting an Overviews of Reviews on health-related topics, it is unclear which combination of bibliographic databases authors should use for searching for SRs. Our goal was to determine which databases included the most systematic reviews and identify an optimal database combination for searching systematic reviews.

**Methods:**

A set of 86 Overviews of Reviews with 1219 included systematic reviews was extracted from a previous study. Inclusion of the systematic reviews was assessed in MEDLINE, CINAHL, Embase, Epistemonikos, PsycINFO, and TRIP. The mean inclusion rate (% of included systematic reviews) and corresponding 95% confidence interval were calculated for each database individually, as well as for combinations of MEDLINE with each other database and reference checking.

**Results:**

Inclusion of systematic reviews was higher in MEDLINE than in any other single database (mean inclusion rate 89.7%; 95% confidence interval [89.0–90.3%]). Combined with reference checking, this value increased to 93.7% [93.2–94.2%]. The best combination of two databases plus reference checking consisted of MEDLINE and Epistemonikos (99.2% [99.0–99.3%]). Stratification by Health Technology Assessment reports (97.7% [96.5–98.9%]) vs. Cochrane Overviews (100.0%) vs. non-Cochrane Overviews (99.3% [99.1–99.4%]) showed that inclusion was only slightly lower for Health Technology Assessment reports. However, MEDLINE, Epistemonikos, and reference checking remained the best combination. Among the 10/1219 systematic reviews not identified by this combination, five were published as websites rather than journals, two were included in CINAHL and Embase, and one was included in the database ERIC.

**Conclusions:**

MEDLINE and Epistemonikos, complemented by reference checking of included studies, is the best database combination to identify systematic reviews on health-related topics.

## Highlights

**What is already known**

- Overviews of Reviews summarize data from several systematic reviews, thus providing a broad picture of relevant evidence.
- Few empirical studies exist that support methodological approaches to producing Overviews of Reviews, including the search process for systematic reviews.

**What is new**

- The present empirical study has shown that in a sample of 1219 systematic reviews, 99.2% of records are included in a combination of the electronic databases MEDLINE via PubMed and Epistemonikos, supplemented by checking reference lists of eligible studies retrieved by these databases.

**Potential impact**

- A resource-efficient search process for systematic reviews should be based on the free-of-charge electronic databases MEDLINE via PubMed and Epistemonikos, supplemented by reference checking.

## Background

Overviews of Reviews (Overviews) are used to summarize data from across multiple systematic reviews (SRs) on the same topic, thus providing a broader picture of evidence relevant for decision-making [1]. Generally, they aim to provide a comprehensive assessment of multiple interventions, populations, or outcomes for a defined health problem using SR-level evidence.

Some methodological components of preparing Overviews are analogous to SRs, such as defining the review question structured into a Population, Intervention, Comparator, Outcomes, and Study types (PICOS) format, setting eligibility criteria, and defining a search strategy, though nuances clearly exist. Other methodological components such as the critical appraisal of included SRs, extraction and analysis of data, and presentation of findings differ more markedly between Overviews and SRs [2–4].

Many specific steps for conducting an Overview are not yet based on empirical research [5], and some steps contain few empirical studies which may guide future recommendations including selection of electronic databases to use when searching for SRs.

Current guidance recommends searching the Cochrane Database of Systematic Reviews (CDSR) to locate Cochrane SRs [1, 6], and a structured search process to locate and select non-Cochrane reviews, (e.g. in MEDLINE [7] and Embase [8]). Guidance also recommends searching additional regional and subject-specific databases (e.g. LILACS [9], CINAHL [10], PsycINFO [11]), major repositories of SRs, and the PROSPERO register [1, 2, 12]. In practice, the majority of Overviews in healthcare-related fields published to date searched at least two databases [13, 14], in addition to reference checking (i.e. looking through the reference list of included studies), and consultation with the relevant Cochrane group [14].

Rathbone et al. have evaluated retrieval of a set of 400 SRs of interventions for hypertension in seven electronic databases [15]. They searched for SRs in the Cochrane Library, DARE, Embase, Epistemonikos, MEDLINE, PubMed Health, and TRIP. For each database, sensitivity, precision, numbers missed, and unique records identified were calculated. Embase had the highest sensitivity (169/400, or 69% of SRs) and retrieved the largest number of unique SR records, i.e. records found by their search in only one database (34/400, or 8.5% of SRs). The authors conclude that searching should not be restricted to one or two major databases, but that all databases should be searched.

In a situation where resources are limited, it remains unclear which combination of electronic resources Overview authors should use for searching for SRs. Therefore, the aims of the present methodological study were: (1) to assess the inclusion of SRs in six electronic databases: MEDLINE [7], CINAHL [10], Embase [8], Epistemonikos [16], PsycINFO [11], and TRIP [17] without limiting the SR topic; (2) to evaluate whether a single database combined with manual reference checking is sufficient to comprehensively retrieve SRs in the context of preparing Overviews; (3) to identify a combination of databases plus reference checking that may be recommended for searching SRs; and (4) to explore possible contexts (for example, for certain Overview types, or for healthcare interventions compared to other fields of healthcare research) in which the recommendation result of objective #3 produces inadequate retrieval of SRs, defined as lower than 95% retrieval [18]. It was not our aim to evaluate search strategies.

## Methods

### Study design

A methodological study was conducted to analyse the rate of inclusion of SRs in six selected electronic resources, and in combinations of two databases with reference checking. We did not write a protocol for this study.

### Search methods

A set of Overviews, including Health Technology Assessment (HTA) reports, Cochrane Overviews, and non-Cochrane Overviews, was obtained from a preceding methodological study, as described in detail in Pieper et al. (2014) [19]. Briefly, a search for overviews was conducted in MEDLINE via PubMed, Embase via Embase.com [8], CINAHL via EBSCOhost [10], PEDro [20], CDSR, DARE, CENTRAL, CMR, HTA, and NHS-EED via the Cochrane Library, and the websites of 127 HTA agencies from inception to May 2012. Search terms were text words related to Overviews and SRs and the search algorithms can be found in Pieper et al. (2014) [19]. The search was limited to Overviews published in English or German between 2009 and 2011.

### Eligibility criteria

Overviews were defined as systematic reviews for which the unit of searching, inclusion and data analysis is the systematic review rather than the primary study [1]. Thus, we included all Overviews that had searched explicitly and systematically for SRs in at least one electronic database, included at least one SR (Overviews including both SRs and primary studies were eligible if the evidence synthesis relied at least in part on SRs, e.g., by combining primary studies and SRs in the evidence synthesis), and critically appraised all included SRs and additional primary studies. A HTA report was defined as “a method of evidence synthesis that considers evidence regarding clinical effectiveness, safety, cost-effectiveness and, when broadly applied, includes social, ethical, and legal aspects of the use of health technologies” [21].

#### Inclusion criteria

- Searched for SRs in at least one electronic database;
- Included at least one SR in their evidence synthesis;
- Critically appraised included SRs and primary studies; and
- Full text publication was available.

#### Exclusion criteria

- Overviews with a methodological focus; and
- Published in a language other than English or German.

The set of included Overviews will be henceforth called the “reference set”.

### Description of electronic databases selected in this study

Six databases were selected to assess inclusion of systematic reviews as described in the section “data collection”, below, namely MEDLINE, CINAHL, Embase, Epistemonikos, PsycINFO, and TRIP. The key features of these databases are described in Table 1.

**Table 1:** Description of electronic databases and resources

	Publisher	Access	Type	Coverage
CINAHL [10]	EBSCO	by subscription	indexed database	nursing, biomedicine, health sciences librarianship, alternative medicine, and allied health topics
Embase [8]	Elsevier	by subscription	indexed database	biomedical literature, 1947 to present
Epistemonikos [16]	Epistemonikos foundation (non-profit)	free of charge	citations database, data scraped from other databases and the web	health evidence, nine supported languages [22, 23]
MEDLINE [7]	U.S. National Library of Medicine (non-profit)	free of charge	indexed database	biomedicine and health literature, 1966 to present [24]
PsycINFO [11]	EBSCO, American Psychological Association	by subscription	indexed database	behavioural science and mental health
TRIP [17]	Trip Inc.	free of charge	clinical search engine	health care [25]

Psyc. topic = mental health- or psychology-related topic

The sources scraped by Epistemonikos include, or have included, CDSR, PubMed, Embase, CINAHL, PsycINFO, LILACS, DARE, HTA database, The Campbell Collaboration online library, JBI Database of Systematic Reviews and Implementation Reports, EPPI-Centre Evidence Library. Updates of algorithms in February and April 2019 have led to an expansion in the dataset by more than a factor of 1.5. TRIP widely collects references from sources of SRs (including Cochrane Library and DARE), guidelines, regulatory agencies (FDA, EMA, NICE, IQWIG), HTA databases, NHS EED, literature databases (PubMed), journals, as well as PROSPERO and clinical trial registries.

### Data collection

From the full text of each included Overview, the following data were extracted into MS Excel (2016): citation, publication title, number of databases searched, name of each database searched, searched in social science/economics databases (yes/no; i.e. EconLit, HEED, NHS EED, IBSS, Social Sciences Citation Index, Social SciSearch, the Campbell Collaboration Database, Social Sciences Abstracts, Social Services Abstracts, Applied Social Science Index and Abstracts, Social Service Information Gateway), searched in additional sources (‘other sources’ yes/no; i.e. reference lists of included studies, queries to experts, Google, Google Scholar, internal departmental files, hand-searching or electronically searching journals, clinical trial or study registries (e.g. clinicaltrials.gov, PROSPERO), publishers’ databases (e.g. Springer, ScienceDirect, Thieme, Wolters Kluwer), HTA agencies’ websites (e.g. https://www.iqwig.de, https://www.dimdi.de, http://www.msac.gov.au)), number of SRs included, Overview type (Cochrane Overview, HTA report, or non-Cochrane Overview), intervention/non-intervention Overview, and mental health- or psychology-related topic (yes/no).

For each Overview, the included SRs were extracted and tagged with the Overview from which they originated. Primary studies were not extracted. HTA reports are usually structured into sections entitled clinical effectiveness, safety, cost-effectiveness, social, ethical, or legal. For HTAs, we only included SRs from the clinical effectiveness section of the report.

The database searches for SRs were performed in April 2019. A stepwise process was followed to identify whether a SR was included in an electronic database, found by reference checking, or included in a database combination:

(A) From the sample of SRs extracted from the Overviews, we determined which of six databases each SR was included in, namely MEDLINE, CINAHL, Embase, Epistemonikos, PsycINFO, or TRIP.
(B) We then determined which database contained the largest overall number of included SRs. This database was identified as the ‘reference database’. We set the reference database to MEDLINE as it had the highest inclusion rate. The SRs included in MEDLINE will be henceforth called the “MEDLINE-included SRs”.
(C) A list of all SRs not included in MEDLINE was compiled. These SRs are called the ‘MEDLINE-non-included SRs’.
(D) For each ‘MEDLINE-non-included SR’ obtained in step C, we then manually checked the reference lists of the ‘MEDLINE-included SRs’ that were cited in the same Overview as the ‘MEDLINE-non-included SR’. The purpose of this step was to find out if each SR not included in MEDLINE could have been identified by reference checking of SRs identified in MEDLINE on the same topic, rather than by additional database searching. The SRs found in the reference lists/bibliographies are henceforth called ‘biblio SRs’.

Finally, we constructed five combined sets of SRs by merging the ‘MEDLINE-included SRs’, ‘biblio-SRs’, and SRs obtained in step A for CINAHL, Embase, Epistemonikos, PsycINFO, and TRIP. For each of these five combined sets, we calculated a combined mean inclusion rate (see statistical analysis). This was done to evaluate whether searching more than one database would expand the study pool.

### Statistical analysis

The dataset generated and analysed during the current study is made available in Additional file 1. For each Overview, we calculated:

(A) the mean inclusion rate (% of included SRs) and corresponding 95% confidence interval (95% CI) separately for each database.
(B) the mean inclusion rate for the reference database (as defined above) combined with reference checking (as described above).
(C) the mean inclusion rates for combinations of the reference database, reference checking, and each of the other five databases.

The Overview-level inclusion rates obtained in statistical analysis steps A to C were then aggregated for the entire dataset by calculating weighted mean inclusion rates and corresponding 95% confidence intervals (95% CI). Weighting was based on the number of SRs included in each Overview.

### Stratification

The goals of stratification were to generate hypotheses on contexts where the results-based recommendations would apply, and to identify situations where retrieval would be inadequate and further searches may be necessary. Inadequate retrieval of SRs was defined as lower than 95% retrieval. Thus, we: (1) investigated whether searching large numbers of databases offers added value, (2) gauged the magnitude of effect when using ‘other sources’ (as defined above in section ‘data collection’), (3) examined whether different Overview types require searching different electronic resources, (4) explored differences in database inclusion between healthcare interventions and other fields of healthcare research, and (5) evaluated the role of specialist databases when such databases exist in the area of the Overview topic, using PsycINFO as an example.

To answer the above objectives (1) to (5), respectively, exploratory analyses were performed for the following strata: (1) number of databases searched (1–3 / ≥4), (2) other sources searched (yes / no), (3) Overview type (Cochrane Overview, HTA report, or non-Cochrane Overview), (4) intervention/non-intervention Overviews, and (5) mental health- or psychology-related topic (yes/no).

Stratification analysis was performed only for strata containing ≥3 Overviews for analysis. For each stratification analysis, the weighted mean inclusion rate with 95% CI was calculated for combinations of the reference database and reference checking with each of the other databases. For analyses with two strata, the weighted difference in means and corresponding *p*-value were calculated using a two-sample weighted t-test (Welch) computed in R version 3.5.1 (2018-07-02) using the R package ‘weights’ [26, 27]. The significance level for each individual test α_i_ was adjusted for multiple testing using the Bonferroni correction, i.e. α_i_ = α_g_/*n* = 0.0017 for a global significance level of α_g_ = 0.05 and *n* = 30 tests (6 databases and 5 stratified analyses).

### Qualitative analysis of missed SRs

All SRs that were not included in a combination of the reference database, reference checking, and the best additional database were analysed qualitatively. Features investigated were the topics of these SRs, whether they were located on websites, included in the other five databases that were investigated in this study, listed in a publisher’s database (e.g. ScienceDirect, Wiley Online Library, Springerlink, De Gruyter), or in Google Scholar.

## Results

### Overviews included in the study

The literature search yielded a reference set of 86 Overviews [28–113], of which 73 had been identified in electronic databases and 13 on HTA websites [19]. The characteristics of the included Overviews are summarised in Table 2, and detailed in Table S1 (see Additional file 2).

**Table 2:** Summary characteristics of *N* = 86 included Overviews

	Median	IQR	Range
Databases searched	6	4–8	1–25
SRs included per Overview	8	5–17	1–103
	n	% (of 86 Overviews)	
Type:			
Cochrane Overview	3	3%	
HTA report	14	16%	
non-Cochrane Overview	69	80%	
	n	% (of 1219 SRs)	
Databases:			
MEDLINE/PubMed^a^	82	95%	
Embase	61	71%	
DARE or Cochrane Library ^b^	59	69%	
CINAHL	36	42%	
PsycINFO	30	35%	
HTA databases (any)	20	23%	
TRIP	3	3%	
Epistemonikos	0	0%	
Any other social science/economics database	18	21%	
Other sources searched	64	74%	
Intervention Overviews	61	69%	
Mental health/psychology-related topic	9	10%	

^a^ MEDLINE also contains all CDSR content [6]. ^b^ DARE was searchable via the Cochrane Library until August 2018, when it was removed along with the other CRD databases NHS EED and HTA, as all three are no longer updated by CRD [114]. HTA is now produced by INAHTA and can be searched via the Canadian Agency for Drugs and Technologies in Health [115]. *IQR* interquartile range

A total of 1219 SRs were included in the 86 Overviews overall, with a median of eight SRs in an Overview. Between one and 25 (median 6) databases had been searched. Among them, the most widely used were MEDLINE and Embase. More than half of the Overviews had searched DARE or the Cochrane Library, which up to 2018 also contained all DARE records. None had searched the international SR-focused database Epistemonikos, which was first launched internationally in August 2012, and was therefore not available at the time of publication of the included Overviews [22].

A wide range of subjects were covered by the included Overviews [13]. Sixty-nine percent were intervention Overviews, defined as Overviews of health-related interventions, i.e. actions taken with the intent of modifying a defined outcome, usually to treat or cure a health condition or change behaviour. Non-intervention Overviews focused, for example, on diagnosis, incidence/prevalence, or risk factors for certain outcomes.

Thirty-five percent (*n* = 30/86) of Overviews searched PsycINFO, but of these, only 30% (*n* = 9/30) had topics related to mental health (i.e. autism, depression, psychosis, or schizophrenia), or psychology (i.e. psychological or psychosocial interventions (such as behavioural therapy)).

### Inclusion in individual databases and database combinations

**Fig. 1:**
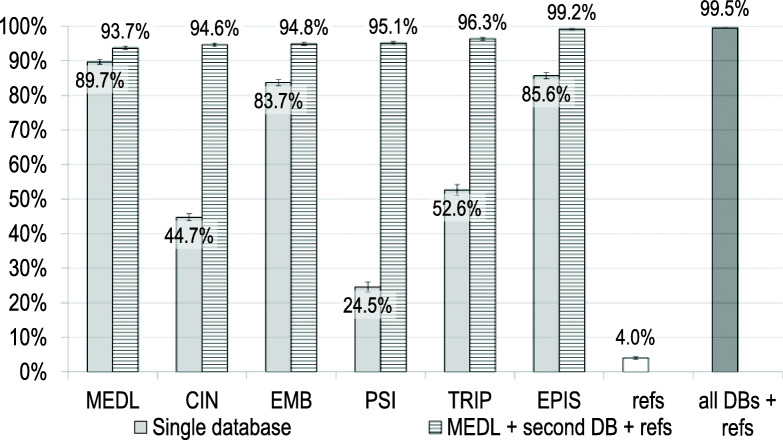
Inclusion rates in individual databases and their combination with MEDLINE and reference checking. MEDL = MEDLINE, CIN = CINAHL, EMB = Embase, PSI = PsycINFO, EPIS = Epistemonikos, DB = database

Among the 1219 SRs retrieved, 90% (*n* = 1093/1219) were included in MEDLINE. The inclusion rates in other databases were lower (Table 3, Fig. 1). Therefore, MEDLINE was used as the reference database.

**Table 3:** Proportion of SRs included in individual databases and their combination with MEDLINE and reference checking

Database	single database % [95% CI]	database + references % [95% CI]	MEDLINE + one database + references; % [95% CI]
MEDLINE	*89.7% [89.0 to 90.3%]*	93.7% [93.2 to 94.2%]	
CINAHL	44.7% [43.7 to 45.7%]		94.6% [94.1 to 94.2%]
Embase	83.7% [82.9 to 84.5%]		94.8% [94.4 to 95.3%]
Epistemonikos	85.6% [84.7 to 86.5%]		*99.2% [99.0 to 99.3%]*
PsycINFO	24.5% [23.1 to 26.0%]		95.1% [94.7 to 95.5%]
TRIP	52.6% [51.0 to 54.1%]		96.3% [95.9 to 96.7%]

Best results in italics

In addition to the SRs included in MEDLINE, a further 4% of SRs (*n* = 49/1219) were identified by reference checking (see Methods, step B). Combinations of MEDLINE and reference checking with the addition of one database (Epistemonikos, PsycINFO, and TRIP) led to inclusion rates above 95% (*n* = 1209/1219, 1159/1219, and 1174/1219, respectively). Among these databases, the inclusion rate was highest in the combination with Epistemonikos (Table 3, Fig. 1).

### Stratification

Table 4 presents the results obtained for each of the strata investigated. For the combination of MEDLINE, Epistemonikos and reference checking, inclusion rates and their 95% confidence intervals were above the 95% retrieval threshold for all individual strata. Therefore, no context was identified in which this combination produced inadequate retrieval of SRs.

**Table 4:** Stratified inclusion rates in database combinations with MEDLINE and reference checking (mean [95% CI])

Stratum	CINAHL	Embase	Epistemonikos	PsycINFO	TRIP
≥4 dB	94.4% [93.8 to 94.9%]	94.5% [93.9 to 95.1%]	*99.3% [99.2 to 99.5%]*	94.8% [94.3 to 95.4%]	96.4% [95.9 to 96.9%]
1–3 dB	95.1% [94.5 to 95.8%]	95.7% [95.1 to 96.4%]	*98.8% [98.4 to 99.2%]*	95.7% [95.2 to 96.3%]	96.0% [95.4 to 96.7%]
Δ in means	0.8%	1.3%	−0.5%	0.9%	−0.4%
*p*-value	0.078	0.004	0.009	0.020	0.370
Other sources	93.5% [92.8 to 94.2%]	93.9% [93.2 to 94.6%]	*98.9% [98.7 to 99.2%]*	94.3% [93.7 to 95.0%]	95.3% [94.7 to 95.9%]
No other sources	96.3% [96.0 to 96.6%]	96.3% [95.9 to 96.6%]	*99.6% [99.5 to 99.7%]*	96.3% [96.0 to 96.5%]	97.9% [97.8 to 98.1%]
Δ in means	2.8%	2.4%	0.7%	1.9%	2.7%
*p*-value	< 0.001*	< 0.001*	< 0.001*	< 0.001*	< 0.001*
HTA report	86.2% [82.8 to 89.6%]	86.2% [82.8 to 89.6%]	*97.7% [96.5 to 98.9%]*	83.9% [80.7 to 87.1%]	95.4% [93.7 to 97.1%]
Cochrane Overview	97.8% [97.5 to 98.1%]	*100.0%*	*100.0%*	97.8% [97.5 to 98.1%]	*100.0%*
Non-Cochrane Overview	95.1% [94.7 to 95.5%]	95.3% [94.9 to 95.7%]	*99.3% [99.1 to 99.4%]*	95.9% [95.5 to 96.2%]	96.2% [95.8 to 96.6%]
Δ in means for HTA vs. non-Cochrane	8.9%	9.1%	1.6%	12.0%	0.8%
*p*-value for HTA vs. non-Cochrane	< 0.001*	< 0.001*	0.012	< 0.001*	0.274
Intervention Overview	95.0% [94.6 to 95.5%]	95.0% [94.6 to 95.5%]	*99.3% [99.1* to *99.5%]*	95.5% [95.1 to 95.9%]	96.0% [95.6 to 96.5%]
Non-intervention Overview	93.2% [92.1 to 94.4%]	94.2% [93.1 to 95.3%]	*98.7% [98.5 to 99.0%]*	93.9% [92.8 to 95.0%]	97.1% [96.7 to 97.6%]
Δ in means	−1.8%	−0.8%	−0.6%	−1.6%	1.1%
*p*-value	0.004	0.182	< 0.001*	0.010	0.001*
Psyc. topic	91.8% [89.8 to 93.8%]	95.9% [94.6 to 97.2%]	*100.0%*	95.9% [94.5 to 97.4%]	93.9% [92.0 to 95.8%]
No psyc. Topic	94.8% [94.3 to 95.4%]	94.7% [94.1 to 95.3%]	*99.1% [98.9 to 99.3%]*	95.0% [94.5 to 95.5%]	96.5% [96.1 to 97.0%]
Δ in means	3.0%	−1.2%	−0.9%	−0.9%	2.6%
*p*-value	0.005	0.093	0.000*	0.243	0.010

Best results in italics; all data are weighted. *Individual comparisons are significant if *p* < 0.0017. Psyc. topic = mental health- or psychology-related topic

#### Databases searched

The Overviews in the reference set had searched for SRs in 1–25 databases. The inclusion rates calculated for Overviews searching 1–3 databases was roughly equal to that calculated for Overviews searching 4 or more databases.

#### Other sources searched

Other sources that were searched by Overview authors include reference lists of included studies, queries to experts, Google, Google Scholar, internal, non-public departmental files from the institutions of Overview authors, hand-searching journals, trial registries, publishers’ databases, journals, institutions, and HTA agencies. Because this represents a larger dataset containing SRs that may not all be included in databases, one may expect that database inclusion rates would be smaller in this stratum.

A total of 74% of Overviews (*n* = 64/86) reported searching at least one other source. In any database combination, the mean inclusion of SRs for Overviews that had searched other sources was only slightly lower than for Overviews that had not searched other sources. For example, the difference was 0.7% for the combination of MEDLINE, Epistemonikos and reference checking.

#### Overview types

**Fig. 2:**
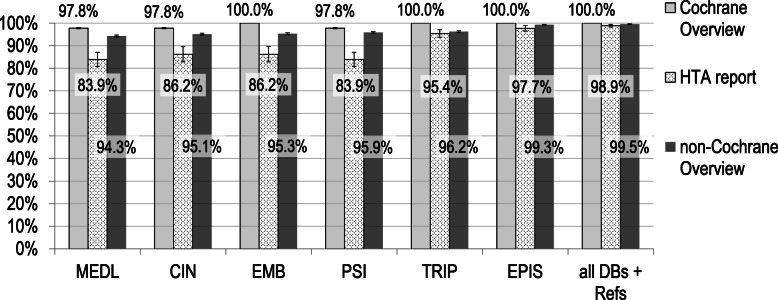
Inclusion rates in database combinations with MEDLINE and reference checking, stratified by Overview type

Overviews were classified into three types, i.e. Cochrane Overviews (*n* = 3), HTA reports (*n* = 14), and non-Cochrane Overviews (*n* = 69). Inclusion of SRs from Cochrane Overviews was complete for combinations of MEDLINE plus reference checking and each of the databases Embase, TRIP, and Epistemonikos (Fig. 2). In contrast, inclusion rates were lower for SRs originating from HTA reports in most database combinations. MEDLINE, Epistemonikos, and reference checking remained the best combination, reaching a mean rate of 97.7% included SRs [96.5 to 98.9]. For non-Cochrane Overviews, which made up 80% (*n* = 69/86) of the total set and contributed 89% (*n* = 1087/1219) of SRs, the results were similar to the results for the total set. Thus, the SR inclusion rate for MEDLINE, Epistemonikos, and reference checking was 99.3% [99.1 to 99.4%].

#### Intervention/non-intervention overviews

Intervention Overviews represented 69% of the overall sample. The difference between SRs originating from intervention vs. non-intervention Overviews was minimal for all databases.

#### Mental health- or psychology-related topics

The present dataset included only 10% (*n* = 9/86) Overviews in the stratum with a mental health- or psychology-related topic, corresponding to 8% (*n* = 98/1219) of included SRs. In this stratum, the inclusion rate in PsycINFO combined with MEDLINE and reference checking was slightly higher than in the stratum without psychology-related topics. However, the combination of the non-specialist databases Epistemonikos and MEDLINE with reference checking was superior, because 100% of SRs with a mental health- or psychology-related topic were included in this combination.

### Qualitative analysis of missed SRs

Ten SRs (*n* = 10/1219, 0.8%) were not identified by the best database combination (Table 5). Of these, one was incorrectly cited in the original Overview [116]. The correct citation [126] was included in the databases CINAHL, Epistemonikos, and Embase. Two were evidence syntheses published on the web only, for which alternative versions were found by database searching [124, 125]. Thus, the evidence for 3/10 missed records would have been included in alternative form, resulting in a total coverage of 99.43% (*n* = 1212/1219) of SRs.

**Table 5:** Qualitative analysis of SRs not included in MEDLINE, Epistemonikos and reference checking

Ref.	Topic	Website	DB Inclusion	Publisher’s DB	Google Scholar
Brunton 2003 [116]	Promoting physical activity amongst children outside of physical education classes	–	–		–
Büssing 2009 [117]	Impact of yoga on chronic pain	–	–	Y (ScienceDirect)	Y
Christopher 1995 [118]	Adolescent Pregnancy Prevention	–	ERIC		–
Clamp 2005 [119]	The value of electronic health records	Y	–		Y1
Erny-Albrecht 2007 [120]	Sublingual immunotherapy in allergic rhinitis and asthma	–	CINAHL, Embase		Y
Evans 2008 [121]	Yoga as treatment for chronic pain conditions	–	CINAHL, Embase	Y (De Gruyter)	Y
Horta 2007 [122]	Evidence on the long-term effects of breastfeeding	Y	–		–
ISMP 2010 [123]	Principles of Designing a Medication Label for Injectable Syringes	Y	–		–
Nelson 2004 [124]	Screening for ovarian cancer	Y	–- ^a^		Y
Shekelle 2009 [125]	Costs and benefits of health information technology	Y	–- ^a^		Y

*DB* database, *ERIC* Education Resources Information Center. ^a^Alternative versions in several databases including Epistemonikos and MEDLINE. Website = Availability of the Overview available on the internet

Three missed references (*n* = 3/1219, 0.24%) were websites not included in any of the databases investigated in the present study [119, 122, 123]. Two missed SRs (*n* = 2/1219, 0.16%) were included in both CINAHL and Embase [120, 121]. One (*n* = 1/1219, 0.08%) was listed on the publisher’s website (ScienceDirect) and found via Google Scholar [117]. Finally, one was not found via any of the routes explored in this study [118]. However, it was included in the Education Resources Information Center (ERIC) database, sponsored by the Institute of Education Sciences of the U.S. Department of Education [127], which had been searched by the Overview authors [93].

## Discussion

We identified MEDLINE/Epistemonikos, complemented by reference checking, as the best combination to retrieve SRs for an Overview of Reviews on health-related topics. This study fill a gap in the evidence around the evaluation of methods in overviews as identified in the MoOR framework by Lunny et al. [5, 128]. Our investigation mapped to the option ‘select the types of databases to search’ in the MoOR framework of all methods used in overviews, and provides clear guidance for authors around which databases to choose when planning an overview.

No single database was found to be sufficiently comprehensive by itself for a systematic search for SRs in the context of preparing Overviews. MEDLINE emerged as the best single source for retrieval of SRs (inclusion rate 89.7%). MEDLINE using the PubMed interface has a good inclusion rate for SRs, and is free of charge and open access.

Among databases assessed in combination with MEDLINE and reference checking (i.e. checking the citation lists of SRs identified in MEDLINE), Epistemonikos gave the highest pooled inclusion rate with 99.2%, followed by TRIP (96.3%). Both Epistemonikos and TRIP are resources specialising in the retrieval of SRs. In spite of the fact that the inclusion rate of SRs was relatively high in Embase (inclusion rate 83.7%), the pooled inclusion rate of MEDLINE, Embase and reference checking was below 95%, possibly due to greater overlap with MEDLINE. The subject-specific databases CINAHL (nursing) and PsycINFO (psychology) had pooled inclusion rates similar to Embase (94.6 and 95.1%, respectively).

Analysis of the different strata did not identify any context in which the combination of MEDLINE, Epistemonikos and reference checking was inadequate.

The difference between the number of SRs retrieved from Overviews that had searched in 4 or more databases was minimal compared to Overviews that had searched in 1–3 databases, casting doubt on the value of searching large numbers of databases without specific justification. When looking at the SRs not covered by our optimal database combination, an additional 0.16% of SRs were included in both CINAHL and Embase, and 0.08% in ERIC. The effect of searching other sources beyond electronic databases (e.g. Google Scholar and publishers’ databases) was found to be similarly small (0.5 and 0.16%, respectively, of studies found in addition to the recommended combination). Nevertheless, a limited number of SRs are published on the web only and not identified by searching the databases investigated in this study (0.24% in this dataset).

The recommended database combination included slightly more SRs from Cochrane and non-Cochrane Overviews than from HTA reports. The better rate of SRs included in Cochrane Overviews (100% of included SRs) is likely a result of guidance for Cochrane Overview authors to search for, and include, only Cochrane SRs [1]. SRs from Cochrane Overviews are more frequently included than from non-Cochrane Overviews (99.3% of included SRs), because all Cochrane Reviews are included in PubMed and other electronic databases [6]. The inclusion rate using the recommended combination was slightly lower for SRs from HTA reports (97.7% of included SRs). This may be because authors of HTA reports typically search more widely, and might thus identify more studies, than authors of Cochrane or non-Cochrane Overviews (median 8 databases searched, compared to 2 databases for Cochrane Overviews and 5 for non-Cochrane Overview; all searches in HTA reports complemented by other methods).

Only one empirical study was found that investigated the types of databases to search [15]. The recall rates in the Rathbone study (88% recall for Cochrane Library and Embase; and 83% for Cochrane Library, Epistemonikos and MEDLINE) are substantially lower than in our study (99.2% indexing in MEDLINE, Epistemonikos and reference checking). Rathbone et al. examined bibliographic database performance in identifying SRs using an approach that was different to ours in several key aspects. First, the study was topic specific and related to interventions for hypertension. Second, the reference set of SRs was generated by searching in the same electronic databases that were later used to analyse retrieval of SRs, and was thus a relative recall analysis based on databases alone [129]. They performed systematic literature searches in seven electronic databases and used search filters for SRs to increase precision. Therefore, their approach primarily evaluated the relative performance of search strategies in bibliographic databases compared to each other, in terms of precision and sensitivity.

In contrast to the Rathbone study [15], our sample was not topic-specific, and included both intervention and non-intervention SRs. We evaluated database coverage independently of the search. Our sample of SRs was larger (1219 vs. 400), and was obtained from published Overviews which had used various methods to identify relevant literature. Our study investigating database inclusion of SRs, as opposed to evaluating the performance of search strategies in different databases.

These methodological differences may explain the different results obtained. In addition, our last search was performed after a major update in the Epistemonikos machine learning algorithms. As a result, a larger number of SRs were included in this resource and retrieved by our search.

Rathbone et al. concluded that “a search of all databases should be performed”. This conclusion is not supported by our results. First, the high database inclusion rate of over 99% that we observed for a combination of two databases and reference checking shows that when a sensitive search strategy is employed, this combination is sufficient for retrieving SRs. Second, in the context of limited resources, it may not be efficient to search additional databases as we found no relevant differences when we stratified inclusion of SRs by Overviews that had searched in 4 or more databases compare with 1 to 3 databases (99.3% vs. 98.8% inclusion, respectively).

### Strengths and limitations of the study

The present methodological study is based on a large sample of 1219 SRs. The SRs were extracted from published Overviews, and were found in diverse electronic databases, often complemented by reference checking, queries to experts, searches of the web (e.g. Google, Google Scholar, government reports), or hand-searching journals, thus making our sample representative of searches in a variety of overviews. We analysed the inclusion rate of SR in databases, and have not used any search strategies for the retrieval of SRs. Therefore, our results are independent of the effectiveness of any search strategy employed.

The results of this study apply only to database coverage. Real-world retrieval of SRs will usually be lower because search strategies can be unreliable and less than 100% sensitive. Another limitation relates to the choice of sample. The lack of recency of the dataset of SRs dated from 1982 to 2011 may be limiting, and future efforts should be made to validate this research in a more up-to-date set of SRs. The reference set of SRs was not generated by hand-searching all relevant resources, which may be considered the gold standard, but relies on relative recall [129]. Some Overview authors may have searched for SRs less extensively or less effectively than others, resulting in a possible bias for studies included in databases. This may affect the absolute values for means and confidence intervals generated within this study. In the worst case, if some Overview authors have missed harder-to-find SRs in their searches, this may tend to even out observable differences between databases and bias the results towards higher overall inclusion rates.

The inclusion of SRs in the six databases is likely to change over time, as the terminology used to describe systematic reviews changes, and as tagging of SRs and algorithms for retrieval of SRs improves. For example, the National Library of Medicine has established a new MeSH publication type “Systematic Reviews” in January 2019, and SRs have retrospectively been re-indexed in MEDLINE [130]. A more general search filter, designed to include SRs that had not yet been indexed, is also available [131]. Other databases may decline in inclusion rates, such as DARE and the NHS EED database (not updated since 2015). The reference set of Overviews was limited to those published in English or German, so that the results are not applicable to SRs published in other languages. In addition, some SRs may have been included in more than one Overview, the effect of which was not assessed. The ‘biblio-SRs’, i.e. those obtained by reference checking in this study, may also have been retrievable by other means. Overview authors were not contacted to clarify the origin of these ‘biblio-SRs’. Also, the results may depend on factors not studied, for example, specific diseases, drugs, or other interventions that are more likely to be included in one database than in another. Furthermore, the present study was not designed primarily to quantify differences between strata, so that the results of the stratification analysis should be considered exploratory. Finally, inclusion in databases was not assessed in duplicate, leading to potential errors in extractions and calculations.

### Future research

Future research should be directed at validating our findings using an up-to-date reference set, assessing whether it is applicable to languages not evaluated in the present study, and exploring the relative contribution of other methods of study retrieval.

The Cochrane Handbook [1] and the Joanna Briggs Institute [132] currently recommend that a structured search process using multiple databases (e.g. MEDLINE and Embase, and additional regional and subject-specific databases, such as LILACS, CINAHL, PsycINFO) be used to retrieve systematic reviews. This guidance will need to be re-evaluated based on our findings that MEDLINE and Epistemonikos, with reference checking of included studies, is the best strategy for retrieval of systematic reviews.

## Conclusion

The present study shows that the literature search for Overviews should be performed in MEDLINE and Epistemonikos, complemented by reference checking.

## Supplementary information

**Additional file 1.** Dataset generated and analysed during the current study.

**Additional file 2: Table S1.** Characteristics of included Overviews.

## Data Availability

All data generated or analysed during this study are included in this published article and its supplementary information files.

## Abbreviations

CI: Confidence interval
DB: Database
HTA: Health Technology Assessment
IQR: Interquartile range
PICOS: Population, Intervention, Comparator, Outcomes, and Study types
SR: Systematic review
