# Supplementary material for: Database combinations to retrieve systematic reviews in overviews of reviews: a methodological study
Source: BMC Med Res Methodol. 2020 Jun 1;20:138. doi: 10.1186/s12874-020-00983-3 (PMC7268249; doi:10.1186/s12874-020-00983-3)
Supplement: Supplementary file 2 — Additional file 2: Table S1. Characteristics of included Overviews. [file 12874_2020_983_MOESM2_ESM.docx]

**Table S1.** Characteristics of included Overviews.

| Overview | Ref. | Type | Names of databases searched | Other sources? | SRs (N) | Intervention Overview? | Mental health /psychology? |
| --- | --- | --- | --- | --- | --- | --- | --- |
| Andersen 2011 | [30] | nCO | PubMed, Embase, CINAHL, Web of Science | Y | 17 | N | N |
| Australia MSAC 2009a | [31] | HTA | Embase, MEDLINE, CDSR, CENTRAL, DARE, NHS EED, HTA, Cochrane Methodology Register | Y | 6 | N | N |
| Australia MSAC 2009b | [32] | HTA | INAHATA, EED, DARE, HTA, CDSR, CENTRAL, Embase, MEDLINE | Y | 4 | N | N |
| Australia MSAC 2010a | [33] | HTA | Embase, MEDLINE, CDSR, CENTRAL, DARE, NHS EED, HTA, Cochrane Methodology Register | Y | 4 | N | N |
| Australia MSAC 2010b | [34] | HTA | Embase, MEDLINE, CDSR, CENTRAL, DARE, NHS EED, HTA, Cochrane Methodology Register | Y | 3 | N | N |
| Australia MSAC 2010c | [35] | HTA | Embase, MEDLINE, CDSR, CENTRAL, DARE, NHS EED, HTA, Cochrane Methodology Register | Y | 6 | N | N |
| Beattie 2011 | [36] | nCO | MEDLINE, CINAHL, BNI & Archive, Embase, DARE, CENTRAL, MRCT, FADE | Y | 5 | Y | N |
| Berkhof 2011 | [37] | nCO | PubMed, PsycINFO, CINAHL, Cochrane Library | N | 11 | Y | N |
| Black 2011 | [38] | nCO | PubMed, Embase, Cochrane Library | Y | 53 | N | N |
| Brouwers 2011 | [39] | nCO | CADTH-EPOC, Health Systems Evidence, McMaster KT+ | N | 34 | Y | N |
| Clar 2010 | [40] | HTA | Cochrane Library, MEDLINE, Embase, PsycINFO, Web of Science | Y | 12 | Y | N |
| Damen 2011 | [41] | nCO | MEDLINE, Embase, ERIC, RDRB Databases | Y | 31 | Y | N |
| De Niet 2009 | [42] | nCO | PubMed, CDSR, DARE | N | 16 | Y | N |
| de Vet 2011 | [43] | nCO | PubMed, Embase, PsycINFO | N | 17 | N | N |
| Diener 2010 | [44] | nCO | PubMed, Embase, CENTRAL | N | 6 | Y | N |
| DIMDI 2010 | [45] | HTA | MEDLINE, Embase, SciSearch, Social SciSearch, AMED, BIOSIS, CAB Abstracts, CCMed, Derwent Drug File, GLOBAL Health, GMS, ISTPB+ISTP/ISSHP, MEDIKAT, ETHMED, INAHTA, DAHTA, DARE, NHS-EED, SOMED, CDSR, CENTRAL | Y | 4 | Y | N |
| DIMDI 2011a | [46] | HTA | CCMed, CDSR, CENTRAL, DAHTA, GMS, GMS Meetings, DARE, INAHTA, SOMED, MEDLINE, MEDIKAT, ETHMED, HECLINET, CAB Abstracts, AMED, NHS-EED, IPA, BIOSIS, Embase, Derwent Drug Backfile, Derwent Drug File, ISTPB+ISTP/ISSHP, SciSearch | Y | 7 | Y | N |
| DIMDI 2011b | [47] | HTA | CCMed, CDSR, CENTRAL, DAHTA, GMS, GMS Meetings, DARE, INAHTA, SOMED, MEDLINE, MEDIKAT, ETHMED, HECLINET, CAB Abstracts, AMED, NHS-EED, IPA, BIOSIS, Embase, Derwent Drug File, ISTPB+ISTP/ISSHP, SciSearch, Social SciSearch | Y | 13 | N | N |
| Ekeland 2010 | [48] | nCO | ACM Digital Library, British Nursing Index, Cochrane Library, DARE, HTA Database, CSA, MEDLINE, Embase, HSTAT, INAHATA, PsycINFO, PubMed, TIE, Web of Science | N | 78 | Y | N |
| Ernst 2010a | [49] | nCO | MEDLINE, Embase, AMED, CINAHL, Cochrane Library, Korean + Chinese DBs (7) | Y | 5 | Y | N |
| Ernst 2010b | [50] | nCO | MEDLINE, Embase, CINAHL, Cochrane Library, AMED, Korean + Chinese DBs (6) | N | 7 | Y | N |
| Ernst 2010c | [51] | nCO | MEDLINE, Embase, AMED, CINAHL, HTA Database, DARE, Cochrane Library, Korean DBs (6), Chinese DBs (2) | Y | 25 | Y | N |
| Ernst 2010d | [52] | nCO | MEDLINE, Embase, AMED, CINAHL, Cochrane Library, Korean + Chinese DBs (7) | Y | 18 | Y | N |
| Ernst 2011a | [53] | nCO | MEDLINE, Embase, AMED, CINAHL, Health Technology Assessments, DARE, Cochrane Library, 5 Korean medical database (Korean Studies Information, DBPIA, Korea Institute of Science and Technology Information, KoreaMed, and Research Information Service System), 1 Chinese database (CNKI) | Y | 9 | Y | Y |
| Ernst 2011b | [54] | nCO | MEDLINE, Embase, AMED, CINAHL, HTA Database, DARE, Cochrane Library, Korean + Chinese DBs (6) | Y | 8 | Y | N |
| Ernst 2011c | [55] | nCO | MEDLINE, Embase, AMED, Cochrane Library | Y | 5 | Y | N |
| Ernst 2011d | [56] | nCO | MEDLINE, Embase, AMED, DARE, CINAHL, Cochrane Library | Y | 4 | Y | N |
| Flodgren 2011 | [57] | Cochrane | CDSR, DARE, TRIP, MEDLINE, Embase, Science Citation Index, Social Science Citation Index, NHS EED, HEED, EconLit, Program in Policy Decision-Making | Y | 4 | Y | N |
| Gibson 2011 | [58] | nCO | CRD Wider Public Health database, CDSR, Criminal Justice Abstracts database, DARE, Campbell Collaboration Database, Evidence for Policy and Practice Information and Coordinating Centre database | Y | 5 | Y | N |
| Greaves 2011 | [59] | nCO | MEDLINE, Embase, CINAHL, PsycINFO, Cochrane Library | Y | 29 | Y | N |
| Heighes 2010 | [60] | nCO | MEDLINE, Embase | Y | 5 | Y | N |
| Hillberg 2011 | [61] | nCO | Campbell Collaboration Database, CRD Wider Public Health database, Cochrane Library, CINAHL, Cambridge Scientific Abstracts, Applied Social Science Index and Abstracts, ERIC, Social Services Abstracts, Sociological Abstracts, Embase, MEDLINE, ScienceDirect, Social Service Information Gateway, SwetsWise, PsycINFO, Zetoc, Web of Science | N | 7 | N | N |
| Hopton 2010 | [62] | nCO | MEDLINE, Allied and Complementary Medicine database, Cochrane Library, Web of Science | Y | 8 | Y | N |
| Huguet 2009 | [63] | nCO | MEDLINE | Y | 2 | Y | N |
| IQWIG 2009a | [64] | HTA | Embase, MEDLINE, CDSR, Dare, HTA | Y | 7 | Y | N |
| IQWIG 2009b | [65] | HTA | Embase, MEDLINE, CDSR, CENTRAL, DARE, SciSearch, HTA, NHS EED, IHTA, HSTAT, TRIP, DAHTA | Y | 11 | N | N |
| IQWIG 2011a | [66] | HTA | MEDLINE, Embase, CDSR, CENTRAL, DARE, HTA | Y | 2 | N | N |
| IQWIG 2011b | [67] | HTA | MEDLINE, Embase, CDSR, CENTRAL, DARE, HTA | Y | 2 | N | N |
| Jepson 2010 | [68] | nCO | CDSR, DARE, AMED, ERIC, CINAHL, Embase, MEDLINE, PsycINFO | N | 103 | Y | N |
| Kamioka 2010 | [69] | nCO | CDSR, PubMed, CINAHL, Web of Science, JDream II, Ichushi-Web | N | 6 | Y | N |
| Kang 2011 | [70] | nCO | PubMed, Cochrane Library, Korean DBs (4) | Y | 16 | Y | N |
| Keus 2010 | [71] | Cochrane | CDSR | N | 3 | Y | N |
| Khambalia 2012 | [72] | nCO | PubMed, MEDLINE, Embase, PsycINFO, CDSR | Y | 8 | Y | Y |
| Kumar 2011 | [73] | nCO | PubMed, CDSR | N | 10 | Y | N |
| Lakke 2009 | [74] | nCO | MEDLINE, CINAHL, Embase, PsycINFO | Y | 9 | N | N |
| LBI 2011 | [75] | HTA | Embase, MEDLINE, Cochrane Library | Y | 6 | Y | N |
| Lee 2011 | [76] | nCO | MEDLINE, Embase, AMED, CINAHL, Cochrane Library, Korean DBs (6), Chinese DBs (2) | Y | 5 | Y | N |
| Lenz 2009 | [77] | nCO | Cochrane Library, PubMed, HTA | Y | 27 | N | N |
| List 2010 | [78] | nCO | PubMed, Cochrane Library, Bandolier | Y | 29 | Y | N |
| Maniglio 2009 | [79] | nCO | AMED, CDSR, EBSCO, ERIC, MEDLINE, PsycINFO, ScienceDirect | Y | 14 | N | N |
| Maniglio 2010 | [80] | nCO | AMED, CDSR, EBSCO, ERIC, MEDLINE, PsycINFO, ScienceDirect | Y | 4 | N | Y |
| Matheson 2010 | [81] | nCO | MEDLINE, Embase, CINAHL, Current Contents, PsycINFO, Cochrane Library | Y | 5 | Y | N |
| Matheson 2011 | [82] | nCO | MEDLINE, Embase, CINAHL, Current Contents, PsycINFO | Y | 24 | N | N |
| Matyas 2011 | [83] | nCO | Embase, MEDLINE, CDSR, Cochrane (HTA), DARE | N | 7 | Y | N |
| Melnik 2010 | [84] | nCO | MEDLINE, CENTRAL, Embase, Lilacs, | Y | 7 | Y | Y |
| Mikton 2009 | [85] | nCO | MEDLINE, PsycINFO, Embase, CINAHL, SSCI, Science Citation Index, LILACS, ERIC, NCJRS, Campbell Library, Cochrane Library, WorldWideScience, KoreaMed, IndMED | Y | 14 | Y | N |
| Minozzi 2010 | [86] | nCO | MEDLINE, Embase, CINAHL | Y | 5 | N | Y |
| Moe 2009 | [87] | nCO | CDSR, DARE, MEDLINE, Embase, PEDro, PsycINFO, CINAHL | Y | 4 | Y | N |
| Monasta 2010 | [88] | nCO | MEDLINE, Embase, Web of Science, CENTRAL, DARE, CINAHL, PsycINFO | N | 22 | N | N |
| Muangpaisan 2010 | [89] | nCO | CENTRAL, MEDLINE, Embase, PsycINFO | Y | 2 | Y | N |
| O'Connor 2009 | [90] | nCO | MEDLINE, CDSR, CENTRAL, DARE, PsycINFO | Y | 28 | N | Y |
| Oestergaard 2011 | [91] | nCO | PubMed, Embase, International Pharmaceutical Abstracts, Web of Science, PsycINFO, Cochrane Library | Y | 19 | Y | Y |
| Onakpoya 2011 | [92] | nCO | MEDLINE, Embase, Cochrane Library, AMED, CINAHL | Y | 9 | Y | N |
| Oxman 2009 | [93] | nCO | CDSR, DARE, Embase, MEDLINE, PubMed | Y | 13 | N | N |
| Palmateer 2010 | [94] | nCO | CINAHL, Cochrane Library, Embase, IBSS, MEDLINE, PsycINFO | Y | 5 | Y | N |
| Peters 2009 | [95] | nCO | PubMed, PsycINFO, ERIC | Y | 55 | Y | N |
| Posadzki 2011a | [96] | nCO | MEDLINE, Embase, AMED, Cochrane Library | N | 44 | Y | N |
| Posadzki 2011b | [97] | nCO | MEDLINE, Embase, Cochrane Library, AMED, CINAHL, PsycINFO | Y | 6 | Y | N |
| Ryan 2011 | [98] | Cochrane | CDSR, DARE | N | 38 | Y | N |
| Safron 2011a | [99] | nCO | PsycINFO, PsycARTICLES, Health Source: Nursing/Academic Edition, MEDLINE, CDSR, ScienceDirect, Ovid databases | Y | 8 | N | N |
| Safron 2011b | [100] | nCO | PsycINFO, PsycARTICLES, Health Source: Nursing/Academic Edition, MEDLINE, CDSR, ScienceDirect, Ovid databases | Y | 17 | Y | N |
| Salvo 2011 | [101] | nCO | MEDLINE, Web of Science, DARE, SCOPUS | N | 29 | Y | N |
| Savard 2011 | [102] | nCO | MEDLINE, Embase, CDSR, DARE, NHS EED, NHS HTA, AgeLine, AMED, SCOPUS, Web of Science, CINAHL | Y | 15 | Y | N |
| Schneider Chafen 2010 | [103] | nCO | PubMed, CDSR, CENTRAL, DARE | N | 1 | N | N |
| Seida 2009 | [104] | nCO | Academic Search Premier, AMED, BIOSIS, Child Development and Adolescent Studies, CINAHL, CDSR, CENTRAL, DARE, Dissertation Abstracts, DOAJ–Directory of Open Access Journals, Embase, ERIC, HTA Database, LILACS, Linguistics and Language Behavior Abstracts, MEDLINE, MEDLINE (in Process and other non-indexed), NHS Economic Evaluation Database, NLM Gateway, OCLC Papers First and OCLC Proceedings, PsycARTICLES, Psychology and Behavioral Sciences Collection, PsycINFO, Social Sciences Abstracts, Web of Science | Y | 12 | Y | Y |
| Smith 2009a | [105] | nCO | PubMed, CINAHL, Cochrane Library | N | 8 | Y | N |
| Smith 2009b | [106] | nCO | MEDLINE, Embase, CINAHL, Cochrane Library, SCI | Y | 22 | Y | N |
| Smith 2010 | [107] | nCO | PubMed, CINAHL, Cochrane Library | N | 6 | Y | N |
| Spearing 2011 | [108] | nCO | PubMed, CINAHL, Embase, PEDro, PsycINFO, EconLit, Lexis, ABI/INFORM, Cochrane Library, AHRQ EPC | Y | 11 | N | N |
| Swinkels 2009 | [109] | nCO | SPORTDiscus, AMED, MEDLINE, Embase, CINAHL, DARE, PEDro, CDSR | Y | 4 | Y | N |
| Tamayo-Velazquez 2010 | [110] | nCO | CDSR, CENTRAL, DARE, NHSEED, HTA Database, MEDLINE, TRIP, CINAHL, PsycINFO, Embase, SSCI, SCI, ERIC, LILACS, CSIC Database, SCOPUS | Y | 7 | Y | N |
| Terry 2012 | [111] | nCO | MEDLINE, PsycINFO, Embase, CINAHL, BNI, AMED, CENTRAL | Y | 5 | Y | N |
| Trudeau 2011 | [112] | nCO | MEDLINE, Embase | Y | 3 | Y | N |
| van Achterberg 2011 | [113] | nCO | PubMed, CINAHL, PsycINFO, CDSR | N | 23 | Y | N |
| van der Feltz-Cornelis 2011 | [114] | nCO | PubMed, Cochrane Library, DARE | Y | 6 | Y | Y |
| Zwicker 2010 | [115] | nCO | AMED, CDSR, CINAHL, DARE, Embase, ERIC, MEDLINE. PEDro, PsycINFO, SPORTDiscus | N | 5 | Y | N |

Ref. = reference; Type = Overview type (HTA, Cochrane overview, or non-Cochrane overview); Other sources? = Have sources other than electronic databases been searched (Y/N); SRs (N) = number of SRs included in Overview; Intervention Overview? = Overviews of health-related interventions (Y/N); Mental health/psychology? = Overview with a mental health- or psychology-related topic (Y/N); nCO = non-Cochrane overview; ERIC = Education Resources Information Center; IndMED = Indexing of Indian Medical Journals; Zetoc = the British Library's table of contents.
